# Decision-Making Competence, Social Orientation, Time Style, and Perceived Stress

**DOI:** 10.3389/fpsyg.2018.00440

**Published:** 2018-04-09

**Authors:** Martin Geisler, Carl Martin Allwood

**Affiliations:** Department of Psychology, University of Gothenburg, Gothenburg, Sweden

**Keywords:** decision making, decision-making competence, perceived stress, social orientation, time approach

## Abstract

Peoples’ decision-making competence, defined as tendency to follow normative rational principles in their decision making, is important as it may influence the extent that requirements are met and levels of perceived stress. In addition, perceived stress could be influenced by social orientation and time style; for example, decisions need to comply with given deadlines and the expectations of others. In two studies, with students (*n* = 118) and professionals (police investigators, *n* = 90), we examined how the three individual difference features: decision-making competence, social orientation, and time approach relate to perceived stress. Results showed that social orientation and time approach were related to levels of perceived stress, but decision-making competence was not. These results indicate that social orientation and time approach are important to consider in relation to perceived stress, but the role of decision-making competence may be less important for perceived stress. However, the role of decision-making competence for perceived stress needs to be further researched.

## Introduction

Stress is an increasing problem for individuals’ health and for society. In work life contexts, including educational settings, a number of factors contribute to perceived stress, for example lack of control over work tasks, time-pressure on performance, and poor feedback and perceived stress appear to be a mediator for negative health outcomes (Mark and Smith, 2008). In addition, various individual difference features, such as decision-making competence, social orientation, and time approach, may contribute to perceived stress. Our definition of decision-making competence follows the prevailing definition in the literature. Thus, decision-making competence is defined as an individual’s tendency to follow normative rational principles in their decision making (e.g., Parker et al., 2017). Decision-making competence is a construct that can be assumed to be related to performance and exhaustion in work life settings (see Ceschi et al., 2017). For example, in a female university sample, Santos-Ruiz et al. (2012) reported that individuals with higher decision-making ability (as measured by the Iowa Gambling Task) had significantly lower levels of cortisol before, as well as after, they were confronted with a stressful situation. In the present study, we investigated how decision-making competence, social orientation, and time approach relate to perceived stress.

In general, decision making does not only include choice but also the processes associated with making a decision, that is, the decision-making process. Various aspects of the decision-making process, not just the final decision, influence decision outcomes (e.g., Keys and Schwartz, 2007). This approach to decision making and decision outcomes is crucial in work life contexts, since many decisions at work are socially embedded (Sanfey, 2007) and have social functions (Tetlock, 2002). At work, people need to be adaptively tuned in to the social environment so that their decision making (process) is responsive to expectations and demands in the social environment (Tetlock, 1985). People who possess this ability are more likely to be efficient at work and therefore less likely to experience that demands will exceed their available resources (Ceschi et al., 2017). Based on this assumption, the present study approaches decision making in a broad way. This is done by including *decision-making competence* as well as *social orientation* and *time approach* (Geisler and Allwood, 2015) among the factors that may influence decision making. In brief, social orientation designates how a person is aware of, relates, and adapts to other people, whereas time approach designates how a person perceives, approaches, and manages time. The rationale for this approach to decision making is that it captures more features of importance for decision making (processes) in applied and complex social settings such as work life and education.

Stress responses can occur when the perceived environmental demands exceed an individual’s regulatory capacity (Koolhaas et al., 2011; see also Karasek, 1979). Stress can come to arise quickly, or evolve over time because of cognitive evaluations of situations and potential consequences (Ursin and Eriksen, 2010). That is, depending on how individuals appraise the balance between perceived resources and perceived demands, stress can be evaluated as challenging or threatening – which in turn have different effects on affect and cognition (Crum et al., 2017). In this regard, ability to make successful decisions is likely to constitute an important aspect of a person’s regulatory capacity essential for such evaluations to be apt and constructive. Research has also found that individuals’ perceived stress can be related to negative health status (e.g., Levenstein et al., 1993; Fliege et al., 2005; Kocalevent et al., 2007; Öhman et al., 2007). Measures of perceived stress assess the level of (threatening or “negative”) stress experienced by an individual and can be assessed in general (e.g., in the last year) or more restricted (e.g., the last month) time periods (Levenstein et al., 1993). As the present research focuses on state aspects of perceived stress, perceived stress was assessed in a recent time period (the last month). We next discuss the three individual difference features suggested to influence perceived stress: decision-making competence, social orientation, and time approach.

### Decision-Making Competence and Perceived Stress

Decision-making research has generally assumed that successful decision making depends on cognitive abilities to perform systematic and normatively rational decision processes. On this basis, Bruine de Bruin et al. (2007; see also Parker and Fischhoff, 2005) developed the *Adult-Decision-Making Competence* battery (A-DMC), collecting tasks that measure the extent that an individuals’ decision making is affected by biases, for example, by the use of heuristics. The initial research showed that A-DMC performance relates to real-life decision-making outcomes (Bruine de Bruin et al., 2007). During the last decade, research has reported that A-DMC performance relates to decision-making styles (Parker et al., 2007; Dewberry et al., 2013), cognitive abilities (Del Missier et al., 2012), risk taking and risk behavior (Weller et al., 2015), school performance (Jacobsson et al., 2012), and financial planning (Parker et al., 2012).

However, research investigating the importance of A-DMC performance in work-life settings is lacking. In fact, only Carnevale et al. (2011) and Geisler and Allwood (2015) have attended to this issue. Carnevale et al. (2011) showed that A-DMC performance in a sample of U.S. high-level leaders outperformed the overall performance reported for Bruine de Bruin et al.’s (2007) U.S. community sample. Furthermore, Geisler and Allwood (2015) found that A-DMC performance in two different professional samples did not contribute to the explanation of reported levels of well-being, experiences of daily hassles, or negative outcomes associated with real-world decision making. Moreover, with regard to the relation between A-DMC and stress, Shields et al. (2016) found that experimentally manipulating acute stress enhanced A-DMC performance. However, the relation between A-DMC and perceived stress has not been studied. The present study contributes by exploring the extent to which A-DMC performance holds predictive validity for perceived stress levels.

### Social Orientation and Perceived Stress

Decision-makers frequently depend on information or contributions from others at various stages of decision processes (Rilling and Sanfey, 2011). Furthermore, decisions often need to be accepted by others in order to achieve successful implementation and reception (Lerner and Tetlock, 1999; Allwood and Hedelin, 2005). Successful decision-makers anticipate these requirements by being attentive to social necessities, tuned in to other people’s reactions, and effectively regulate and adjust decision-making processes accordingly (Ceschi et al., 2017). Indeed, research has shown that social orientation (e.g., self-monitoring, empathy, and emotional intelligence) has an effect on decision-making performance (see e.g., Telle et al., 2011; Geisler and Allwood, 2015; Ramsøy et al., 2015).

As indicators of social orientation contributing to decision making, we measured individual differences in *self-monitoring, Machiavellian personality*, and *trait-emotional intelligence* (TEI). Self-monitoring reflects self-reported sensitivity to recognize subtle hints in social interactions, and to be able to modify one’s behavior accordingly (Gangestad and Snyder, 2000). Self-monitoring has been reported to be related to successful and adaptive functioning in working life, for example, positively related to job performance and promotion (Day et al., 2002). Machiavellian personality refers to tendencies of an insidious, deceitful, and manipulative approach to other people. Machiavellianism is related to, yet differentiated from, offensive personality constructs as sub-clinical narcissism and psychopathy (Paulhus and Williams, 2002). Research has shown that Machiavellian tendencies are negative in social and working-life settings since individuals high in Machiavellianism are more prone to make egoistic and amoral decisions (Dahling et al., 2009). Finally, TEI refers to the disposition to be tuned in to, and able to regulate emotional reactions in self and others (Petrides and Furnham, 2001). TEI relates to decision-making success in work-life settings (Mikolajczak et al., 2012), and coping with stress in the form of needed “emotional labor,” that is, the need to handle the clash between one’s “real” subjective feelings and socially required feelings (Mikolajczak and Luminet, 2008).

### Time Approach and Perceived Stress

How people perceive and approach time affect their decision making (Wittman and Paulus, 2007). As time approach guides people’s judgments and decisions, it is an important feature to consider in working life (Gupta et al., 2012). One way to define and measure individual differences in the approach and management of time and time-related activities is to attend to time-styles (Usunier and Valette-Florence, 2007), which basically reflect aspects of engagement in the decision process. Time-styles relate to decision making hands on; for example, the extent to which one values and structures time or the extent to which one succumbs to given time restrictions. Moreover, differences in time styles can be seen to reflect essential aspects of the extent to which people are committed to and engaged in their decision-making processes. Previous research have reported that individual differences in how time is perceived and managed is related to various aspects of well-being (Drake et al., 2008) and self-reported stress (Claessens et al., 2007). Therefore, differences in time-styles are likely related to levels of perceived stress. Furthermore, the present research also included differences in procrastination. Procrastination is the tendency to postpone the commencement or completion of intended tasks (Lay, 1986). With regard to self-reported stress, procrastinators have been found to experience short-term benefits but long-term costs (Tice and Baumeister, 1997). Hence, the present study measured *time-styles* (Usunier and Valette-Florence, 2007) and *procrastination tendencies* (Lay, 1986) as features of individual differences in time approach.

### The Present Study

The present study investigated how three individual difference features assumed to be important for successful decision making: *decision-making competence social orientation*, and *time approach*, contribute to the explanation of *perceived stress*. Based on the research reviewed above (e.g., Bruine de Bruin et al., 2007; Santos-Ruiz et al., 2012), *Hypothesis 1* expected that higher A-DMC performance would be associated with lower levels of perceived stress. Furthermore, *Hypothesis 2* expected that social orientation would provide a unique amount of explained variance for perceived stress. Specifically, higher reports of self-monitoring and TEI were expected to be associated with less perceived stress, whereas higher reports of Machiavellian tendencies were expected to be associated with more perceived stress. Finally, *Hypothesis 3* expected that time approach would provide unique explained variance in perceived stress. Reports of time styles characteristic for an engaged time approach were expected to be related to less perceived stress, whereas reports of time styles reflecting a non-engaged time approach, and higher reports of procrastination, were expected to be related to more perceived stress.

The present study included two samples: university students (study 1) and police investigators (study 2). These specific samples were targeted since decision making and perceived stress are characteristic features in the daily work of both students and police investigators (Kop et al., 1999; Abdollahi, 2002; Deniz, 2006).

## Materials and Methods – Study 1

### Procedure

This research was approved by the Regional Ethical Review Board, Gothenburg secretariat (Sweden), 2011-02-21, dnr: 071-11. In sum, 118 Swedish university students participated (85% women, mean age = 25.8 years, *SD* = 4.8). Participants were recruited at lectures or by e-mail invitations and compensated with a movie-ticket and a lottery-ticket (approx. total value of 15 USD). Written informed consent was obtained from all participants (studies 1 and 2). The data were collected in sessions of 1–15 participants in a large computer room. Participants completed the web-based questionnaire individually. The time for participation was 40–60 min.

### Materials

Tests and scales unavailable in Swedish were translated by conventional back-translation procedures: A-*DMC Self-Monitoring Scale* (SMS), *Machiavellian Personality Scale Procrastination scale*, and *Time-Style Scale* (TSS). A-DMC was used to measure decision-making competence, whereas the SMS and the Machiavellian personality scale were used to measure social orientation, and the procrastination scale and the TSS were used to measure time approach.

#### Adult-Decision-Making Competence Battery (A-DMC)

The A-DMC (Bruine de Bruin et al., 2007) includes six components. Scores are calculated in terms of internal consistency and/or accuracy for the components: *Resistance to Framing* (RF), *Applying Decision Rules* (ADR), *Consistency in Risk Perception* (CRP), *Under/Overconfidence* (UOC), *Resistance to Sunk Costs* (RSC), and *Recognizing Social Norms* (RSN). The component RF measures consistency as observed over two different sets of framing tasks; attribute framing tasks and risky-choice framing tasks. The ADR measures the extent to which individuals are able to follow decision-rules of different complexity, whereas CRP concerns ability to correctly judge probability. Next, the component UOC measures the ability to recognize the correctness of one’s own knowledge. RSC deals with the ability to ignore prior investments (costs or efforts). Finally, RSN measure the ability to assess social norms. An individual’s overall performance is indicated by the A-DMC index, calculated as the unweighted average of the individual’s standardized scores for each of the six components (for a detailed description of the A-DMC, see Bruine de Bruin et al., 2007).

Two A-DMC components were adjusted due to cultural differences between the United States and Sweden (Weller et al., 2015). For RSN, 6 of 16 items were excluded as they were considered inappropriate in a Swedish setting. The adjusted A-DMC was pilot-tested (*N* = 15, 66% women, mean age = 24.4 years), and demonstrated good reliability for the amended RSN (α = 0.73, cf. α = 0.64 in Bruine de Bruin et al., 2007). One item in the component RSN showed no variation in the pilot study and was therefore excluded. Moreover, because of concerns raised by pilot-study participants that certain UOC items were inappropriate in Swedish settings, 10 of 34 items were replaced. A full list of amendments in A-DMC questions is reported in the Supplementary Material.

#### Self-Monitoring Scale (SMS)

We used the revised SMS with 13 items (Lennox and Wolfe, 1984), which has been found reliable (Day et al., 2002). Items are rated on 6-point Likert-type scales, with scores calculated for the total scale or divided into the two subscales: *ability to modify self-presentation* and *sensitivity to expressive behavior of others*. An example item from the *ability to modify self-presentation* subscale is “*Once I know what a situation calls for, it’s easy for me to regulate my actions accordingly.*” Cronbach’s alpha was α = 0.84 for *ability to modify self-presentation* and α = 0.73 for *sensitivity to expressive behavior of others.*

#### Machiavellian Personality Scale (MPS)

The MPS has 16 items rated on 5-point Likert-type scales (Dahling et al., 2009). Ratings are calculated for total score or divided into the four subscales: *amoral manipulation desire for control desire for status*, and *distrust of others*. An example item from the *distrust of others* subscale is “*I dislike committing to groups because I don’t trust others*” (α = 0.90).

#### Procrastination Scale (PS)

The PS (Lay, 1986) has 20 items rated on 5-point Likert-type scales. An example item is “*I generally return phone calls promptly*” (reversed scoring, α = 0.87).

#### Time-Style Scale (TSS)

The 29 items of the TSS (Usunier and Valette-Florence, 2007) are rated on 7-point Likert-type scales and make up four time-style dichotomies: *preference for economic time* (e.g., schedule and structure time and attend to one task at a time) – *preference for non-organized time* (e.g., to not schedule one’s time and to attend to multiple tasks simultaneously), *orientation toward the future* (e.g., to focus on the future) – *orientation toward the past* (e.g., focus on the past and be nostalgic), *time submissiveness* (e.g., a dutiful and conforming approach to time) – *time anxiety* (e.g., being uncomfortable and experiencing adjustment problems when faced with time-related activities), and *tenacity* (e.g., delay of gratification) – *preference for quick return* (e.g., be impulsive). Based on the four style dichotomies and to reduce complexity, two time-style indexes were calculated: an engaged time-style index and a non-engaged time-style index. The engaged time-style index collected the time-styles: economic, orientation toward the future, time submissiveness, and tenacity. The non-engaged time-style index collected the time styles: non-organized, orientation toward the past, time anxiety, and preference for quick return. Analyses of Cronbach’s alpha supported the internal consistency among items for the respective index (engaged time style, α = 0.81; non-engaged time style, α = 0.78). The two indexes were then calculated by the same basic procedure used for the A-DMC index; individuals’ scores are calculated by the unweighted average of standardized scores for each of the respective four time styles. Example item from the time-style *tenacity*: “*When I am interrupted doing a task, I almost always go back to it as soon as I can.*”

### Perceived Stress Questionnaire (PSQ)

The PSQ assesses stress-related symptoms (Levenstein et al., 1993). We used a validated Swedish version of the PSQ (Bergdahl and Bergdahl, 2002). Building on previous research (Salo and Allwood, 2011; Allwood and Salo, 2012), we used a shortened PSQ-version shown to be reliable (α = 0.90) including 17 of the original 30 items. The respondents rated each item with respect to the degree of occurrence during the last month on a four-point rating scale with the alternatives “*Almost never*,” “*Sometimes*,” “*Often*,” and “*Usually*.” An example item is “*You have trouble relaxing.*”

Previous research has consistently showed PSQ to have Cronbach’s alpha around 0.90. With respect to validity, Kocalevent et al. (2007) in a large sample, population-based, study reported that PSQ shows discriminant validity in that it correlates moderately with neuroticism (*r* = 0.48) and that results from previous studies show that PSQ correlates between 0.75 and 0.54 for trait anxiety and between 0.18 and 0.40 for state anxiety, depending on the form of PSQ and study. Research by Fliege et al. (2005), Kocalevent et al. (2007), and Öhman et al. (2007) demonstrate that PSQ also shows other forms of validity such as predictive and convergent validity.

## Results

**Table 1** presents the descriptive statistics. Scales and subscales showed appropriate levels of reliability and variance. As the A-DMC index is defined by separate components that measure relatively distinct decision-making processes, and an individual may excel in performance on one component but not in others (Bruine de Bruin et al., 2007), level of alpha reliability is not relevant to consider for this index.

**Table 1:** Descriptive statistics for study 1 (*N* = 118) and study 2 (*N* = 90).

	Study 1	Study 2	Study 1	Study 2	Possible range	Study 1	Study 2
Component	*M* (*SD*)	*M* (*SD*)	Skewness	Skewness		Observed range	Observed range
**Decision-making competence**							
A-DMC index^b^	0.0 (0.51)	0.0 (0.52)	–0.744	–0.200	–	–2.00 to 0.92	–1.19 to 1.17
**Social orientation**							
SMS ability to modify self-presentation	30.25 (5.57)	27.66 (4.43)	–0.153	–0.523	7–42	17–42	17–35
SMS sensitivity to expressive behavior of others	25.61 (4.19)	25.20 (4.07)	0.008	–0.165	6–36	15–36	14–35
Machiavellian Personality Scale	38.43 (11.01)	–	0.656	–	16–80	21–71	–
Trait-emotional intelligence (Global)	–	156.58 (18.76)	–	–0.010	30–210	–	115–204
**Time approach**							
Procrastination	60.45 (12.77)	43.36 (10.68)	–0.387	0.144	20–100	29–88	25–74
Engaged time-style index^b^	0.00 (0.65)	0.00 (0.65)	–0.301	–0.725	–	–1.65 to 1.45	–2.63 to 1.56
Non-engaged time-style index^b^	0.00 (0.61)	0.00 (0.54)	0.374	0.224	–	–1.23 to 1.69	–1.26 to 1.67
**Outcome**							
Perceived Stress Questionnaire^c^	2.21 (0.61)	1.89 (0.49)	0.350	0.599	1–4	1.12–3.71	1.03–3.63

*A-DMC index, Adult-Decision-Making Competence index; SMS, Self-Monitoring Scale; TSI, Time-Style Index. ^*a*^*Cronbach’s alpha.*^*b*^Mean score is zero as the A-DMC index is calculated by the unweighted average of the individual’s standardized scores for each of the six components. ^*c*^Perceived Stress Questionnaire: Study 1 used a 17-item version (Salo and Allwood, 2011).*

### Correlations

**Table 2** shows the correlations. Age was positively related to A-DMC index, but A-DMC index was not significantly correlated to *perceived stress* (PSQ). The correlation between A-DMC index and PSQ was *r* = -0.154, *p* = 0.086. We also controlled for the correlation when the A-DMC index only included the unmodified A-DMC components (i.e., RF, ADR, CRP, and RSC), the correlation between A-DMC index and PSQ was then: *r* = -0.171, *p* = 0.064. The two time-style indexes were negatively related. Furthermore, Machiavellianism and both time styles indexes were found to be positively related to PSQ.

**Table 2:** Correlations for study 1.

	1	2	3	4	5	6	7	8	9
1. Age	–								
2. A-DMC	0.221*	–							
3. SMS ability	–0.135	–0.109	–						
4. SMS sensitivity	0.250**	0.058	0.349**	–					
5. MPS score	–0.107	–0.318**	0.179	0.184*	–				
6. Engaged TSI	–0.119	–0.174	0.127	–0.063	0.114	–			
7. Non-engaged TSI	–0.053	0.024	–0.225*	0.027	0.189*	–0.249**	–		
8. Procrastination	–0.033	–0.023	–0.163	–0.039	0.082	–0.372**	0.419**	–	
9. Perceived stress	–0.029	–0.159	–0.077	0.099	0.228*	0.309**	0.256**	0.155	–

*The presented significances are for Pearson’s correlation and two-tailed tests of significance. A-DMC, Adult-Decision-Making Competence index; SMS, Self-Monitoring Scale; MPS, Machiavellian Personality Scale; TSI, time-style index. ^∗^*p* < 0.05. ^∗∗^*p* < 0.01.*

### Regression Analyses

To test the hypotheses, hierarchical multiple regression analyses were performed (**Table 3**). *Step 1* controlled for the effect of gender and age. The consecutive regression blocks were built by A-DMC index (*Hypothesis 1*) in *Step 2* and social orientation and time approach measures (*Hypothesis 2* and *Hypothesis 3*) tested in two separate versions of *step 3* and *step 4*. Altering the order of social orientation and time approach in step 3 and step 4 controlled for the unique contribution provided by these two features of decision-making skills.

**Table 3:** Hierarchical Regression of Perceived Stress – Study 1.

	Total *R*^2^	Adjusted *R*^2^	Δ*R*^2^	Test of Δ*R*^2^
Model A				
Step 1: Gender and age	0.001	–0.017	0.001	*F*(2, 115) = 0.048, *p* = 0.953
Step 2: A-DMC	0.025	0.000	0.024	*F*(1, 114) = 2.85, *p* = 0.094
Step 3: Social orientation	0.092	0.043	0.067	*F*(3, 111) = 2.72, *p* = 0.048
Step 4: Time approach	0.287	0.227	0.195	*F*(3, 108) = 9.84, *p* < 0.001
Model B				
Step 3: Time approach	0.257	0.217	0.232	*F*(3, 111) = 11.54, *p* < 0.001
Step 4: Social orientation	0.287	0.227	0.030	*F*(3, 108) = 1.51, *p* = 0.216

*Models A and B differ with respect to the order in which social orientation and time approach were entered into the model in steps 3 and 4. A-DMC, Adult-Decision-Making Competence index; social orientation, Self-monitoring scale; Machiavellian Personality Scale; time approach, engaged time-style index; non-engaged time-style index, procrastination scale.*

Gender and age were not found to have an effect on PSQ. *Hypothesis 1* was not confirmed. A-DMC index was not significantly related to PSQ. However, when social orientation (*Hypothesis 2*) was inserted in step 3 of the model the contribution of this block was significant (*p* = 0.048, *R*^2^ change = 7%). However, none of the single social orientation facets were found to be significant single predictors. Moreover, when social orientation was inserted in step 4 of the model, this step was not close to significant.

Time approach clearly contributed to PSQ, confirming *Hypothesis 3*. When measures of time approach were inserted in step 3, a significant 23% variance in PSQ was explained. Here, all three facets of time approach, that is, engaged time-style index (β = 0.446, *p* < 0.001), non-engaged time-style index (β = 0.238, *p* = 0.014), and procrastination (β = 0.199, *p* = 0.039) were found to be related to perceived stress. Moreover, when time approach was inserted in step 4, it provided a significant 20% explained variance in PSQ. Again, all three facets of time approach were significant predictors: Engaged time-style index (β = 0.446, *p* < 0.001), non-engaged time-style index (β = 0.238, *p* = 0.014), and procrastination (β = 0.199, *p* = 0.039).

## Materials and Methods – Study 2

### Procedure

After initial contact with management officials, police investigators were randomly selected over different areas of operations (e.g., driving offenses and violent crimes) and geographic stations (e.g., urban/rural) by collecting email addresses for 165 police investigators. The web-based questionnaires were answered by 66 participants (participation rate = 40%), but 21 questionnaires were incomplete and therefore excluded, leaving 45 participants. However, it turned out that the electronic invitations had failed to reach all presumptive participants, as some email addresses were not activated for external communication. To facilitate participation, additional police investigators were invited to participate by paper-and-pen questionnaires. Hence, based on the same criteria and procedure as before, an additional randomized selection of police investigators was performed. A total of 195 invitations were sent out by paper-and-pen questionnaires and 50 participants answered (participation rate = 26%, total participation rate = 32%). For the paper-and-pen questionnaire, five questionnaires were incomplete and excluded. Moreover, for the paper-and-pen questionnaires, there was a limited concern of missing data (37 cases of missing data for 36 items). Missing data analysis showed no pattern; thus, data were considered to be missing at random and replaced by computations using the Expectation–Maximization method (Kline, 2005). Accordingly, in the final sample of 90 participants (37% women, mean age 46 years, *SD* = 11.21), 45 participants had answered the web-based questionnaire and 45 participants the paper-and-pen questionnaire. There were no obvious differences between the two sub-samples and all participants were instructed to answer the questionnaire-battery individually by their own desk. The time for participation was 30–50 min.

### Materials

In study 2, the A-DMC component UOC was excluded. This was because, first, the time available for participation was limited for the professional sample of study 2. The A-DMC is a time-consuming measure and the UOC is especially time-consuming (see e.g., Weller et al., 2015). Second, previous research has questioned the importance of the UOC (e.g., Carnevale et al., 2011). Moreover, three of the nine items on the A-DMC component RSN were replaced. The reason for this was based on the consideration that the three items would be considered odd for the police sample since they asks, “if it is sometimes OK” to break the law (i.e., to steal, to commit a crime which could put you in jail, to experiment with marijuana). A full list of amendments in A-DMC questions is reported in the Supplementary Material. Previous research has used shortened versions of the A-DMC or only attended to certain components (e.g., Carnevale et al., 2011).

Study 2 used the adult version of the procrastination scale (Lay, 1986; α = 0.82). Furthermore, the full-length version of the PSQ (Levenstein et al., 1993; Bergdahl and Bergdahl, 2002) was used, since the focus on perceived stress was of specific interest for the police organization (α = 0.94). Moreover, measuring Machiavellian tendencies among police investigators could have evoked suspicion and resistance, but study 1 demonstrated that Machiavellian tendencies were associated with perceived stress (**Table 2**). Therefore, study 2 instead investigated if a relation also could be found for individual differences in the reverse tendencies. As dispositions measured by the TEI Questionnaire (TEIQue) and Machiavellianism (i.e., egoistic, amoral, and distrustful) have been found to be essentially opposite (Jones and Paulhus, 2009; Petrides et al., 2011), we used the TEIQue. Moreover, as in study 1, the SMS was used (ability to modify self-presentation, α = 0.74, sensitivity to expressive behavior of others, α = 0.80). Finally, with respect to the TSS analyses of Cronbach’s alpha again supported the internal consistency among items for the respective index (engaged time style, α = 0.83, non-engaged time style, α = 0.75).

#### Trait-Emotional Intelligence Questionnaire – Short-Form (TEIQue-SF)

The TEIQue comprehends important aspects of decision making in social contexts, such as the ability to adequately recognize emotions and exhibit emotional adaptability (Telle et al., 2011; Mikolajczak et al., 2012). Due to work-load considerations, we used the short-form (the TEIQue-SF) that provides a global TEI score (Petrides and Furnham, 2006). The short-form comprises 30 of the 153 items from the full-version (Petrides and Furnham, 2001). As a Swedish translation of the TEIQue-SF was unavailable (nor of the TEIQue), the TEIQue-SF was back-translated (α = 0.80). An example item is: “*Many times, I can’t figure out what emotion I’m feeling.*”

## Results

**Table 1** presents the descriptive statistics. The A-DMC index, the engaged and the non-engaged time-style indexes were computed by the use of *z*-transformations for the respective components or scales. For the social orientation measures, the present sample showed overall lower self-reports of *Self-Monitoring*, compared to the student sample in study 1. Specifically, the samples differed on the *ability to modify self-presentation* as students reported overall higher levels of this ability (*M* = 30.25, *SD* = 5.57), compared to police investigators (*M* = 27.67, *SD* = 4.43), *t*(206) = -3.72, *p* < 0.001. For time approach, *procrastination* tendencies were lower (*M* = 43.36, *SD* = 10.68) compared to study 1 (*M* = 60.45, *SD* = 12.77), *t*(206) = -8.37, *p* < 0.001. In addition, the present sample’s level of perceived stress (*M* = 1.89, *SD* = 0.49) was lower compared to study 1 (*M* = 2.21, *SD* = 0.61), *t*(206) = -4.16, *p* < 0.001.

### Correlations

Correlations are presented in **Table 4**. The A-DMC index was not related to PSQ or any other measure. The correlation between A-DMC index and PSQ was *r* = -0.146, *p* = 0.171. As in study 1, we also controlled for the correlation if the A-DMC index only included the unmodified A-DMC components (i.e., RF, ADR, CRP, and RSC), the correlation between A-DMC index and PSQ was then: *r* = -0.049, *p* = 0.647. Reports of TEI showed a high, negative correlation with PSQ as well as with reports of procrastination and the non-engaged time-style index. Moreover, both procrastination and the non-engaged time-style index were positively related to PSQ.

**Table 4:** Correlations for study 2.

	1	2	3	4	5	6	7	8	9
1. Age	–								
2. A-DMC	–0.057	–							
3. SMS ability	–0.311**	–0.063	–						
4. SMS sensitivity	–0.186	–0.182	0.326**	–					
5. TEIQue	–0.127	–0.001	0.274**	0.114	–				
6. Engaged TSI	–0.102	–0.152	0.117	0.125	0.144	–			
7. Non-engaged TSI	0.095	–0.193	–0.001	0.057	–0.455**	–0.103	–		
8. Procrastination	–0.179	–0.108	–0.102	0.022	–0.423**	–0.536**	0.268*	–	
9. Perceived stress	–0.252*	–0.146	0.189	0.188	–0.405**	0.039	0.397**	0.341**	–

*The presented significances are for Pearson’s correlation and two-tailed tests of significance. A-DMC, Adult-Decision-Making Competence index; SMS, Self-Monitoring Scale; TEIQue, Trait-Emotional Intelligence Questionnaire; TSI, time-style index. ^∗^*p* < 0.05. ^∗∗^*p* < 0.01.*

### Regression Analyses

Hierarchical multiple regression analyses were performed to test the hypotheses (**Table 5**). *Step 1* controlled for gender and age and *step 2* controlled for type of survey (dummy-coded as, online = 0; paper-and-pen = 1). The succeeding regression blocks were built as in study 1, inserting the A-DMC index in *step 3* (*Hypothesis 1*). In order to test *Hypothesis 2* (the added and unique contribution of social orientation) and *Hypothesis 3* (the added and unique contribution of time approach), separate analyses were performed in which these measures were used in *step 4* and *step 5*, respectively.

**Table 5:** Hierarchical Regression of Perceived Stress – Study 2.

	Total *R*^2^	Adjusted *R*^2^	Δ*R*^2^	Test of Δ*R*^2^
Model A				
Step 1: Gender and Age	0.064	0.040	0.064	*F*(2, 79) = 2.69, *p* = 0.074
Step 2: Survey	0.103	0.069	0.040	*F*(1, 78) = 3.45, *p* = 0.067
Step 3: A-DMC	0.117	0.072	0.014	*F*(1, 77) = 1.23, *p* = 0.271
Step 4: Social orientation	0.357	0.296	0.239	*F*(3, 74) = 9.18, *p* < 0.001
Step 5: Time approach	0.409	0.326	0.053	*F*(3, 82) = 2.11, *p* = 0.107
Model B				
Step 2: Time approach	0.340	0.277	0.222	*F*(3, 74) = 8.31, *p* < 0.001
Step 3: Social orientation	0.409	0.326	0.069	*F*(3, 71) = 2.78, *p* = 0.047

*Models A and B differ with respect to the order in which social orientation and time approach were entered into the model in steps 2 and 3. A-DMC, Adult-Decision-Making Competence index; social orientation, Self-Monitoring Scale; Trait-Emotional Intelligence Questionnaire; time approach, engaged time-style index, non-engaged time-style index, procrastination scale.*

Gender and age (*step 1*) or type of survey (*step 2*) was not found to have a significant effect on PSQ. In step 3, A-DMC index was not significantly related to perceived stress, not providing support for *Hypothesis 1.* However, when social orientation was inserted in step 4 of the model, 24% of the variance in perceived stress was explained. Reports of TEI (β = -0.514, *p* < 0.001) were the significant predictor. When social orientation was inserted in step 3, the contribution of this block was lower, 7%, but significant (*p* = 0.047) – and reports of TEI (β = -0.322, *p* = 0.014) were still a significant predictor.

The regression analyses also confirmed *Hypothesis 3*. When time approach was inserted in step 4, a significant 22% of the variance in perceived stress was explained. Two of the three facets of time approach were significant predictors: non-engaged time-style index (β = 0.363, *p* = 0.001) and procrastination (β = 0.300, *p* = 0.020). When time approach was inserted in step 5, the contributed explanation of this block was not significant. However, in step 5, the non-engaged time-style index (β = 0.222, *p* = 0.048) was still found to be a significant predictor.

## Discussion

This study investigated the extent that three individual difference variables assumed to contribute to decision making: decision-making competence, social orientation, and time approach, predict levels of perceived stress in a student sample and in a sample of professionals (police investigators).

### Decision-Making Competence

The results from study 1 and study 2 did not render support for *Hypothesis 1*, stating that individual differences in decision-making competence (i.e., A-DMC performance) would be associated with levels of perceived stress. No association between A-DMC performance and perceived stress was found. Thus, the general benefits associated with A-DMC performance previously reported (e.g., Bruine de Bruin et al., 2007) did not generalize to the stress domain in the present study. This may be explained by the more homogeneous samples targeted in the present research (students and professionals, cf. the community sample in Bruine de Bruin et al., 2007). A further reason why A-DMC did not relate to perceived stress is that evaluations of demands and resources (Koolhaas et al., 2011) and the cognitive activation of stress responses (Ursin and Eriksen, 2010) are processes that are based on subjectively perceived levels and considerations, whereas A-DMC is a performance measure. That is, it is not known, and should be explored in future research, to what extent individuals are aware of their own decision-making competence level and if such awareness relates to perceived stress.

Moreover, although acute stress has been reported to enhance A-DMC performance (Shields et al., 2016), as noted, a relation between A-DMC and perceived stress was not observed in the present research. A difference between the studies is that the present research attended to (subjective) perception of negative stress, whereas Shields et al. (2016) experimentally manipulated acute stress. In addition, although we measured perceived stress in a recent and restricted time period (i.e., the last month), this indication of stress may be considered as more reflective of chronic stress compared to measures of acute stress. In brief, the present results do not provide support for the suggestion that decision-making competence constitutes a coping resource for perceived stress (Santos-Ruiz et al., 2012). It is possible that further research might show a relationship between decision-making competence and perceived stress under certain conditions (e.g., in larger and more heterogeneous samples) – but our results suggest that a general relation should not necessarily be expected in work-life settings.

### Social Orientation

The results supported the assumption that individual differences in social orientation influence perceived stress (*Hypothesis 2*). The contribution of social orientation did reach significance in study 1, but the contribution was more substantial in study 2. A possible reason for this difference is that study 1 included a measure of Machiavellianism, whereas this measure was replaced by a measure of TEI in study 2. Although Machiavellian tendencies were found to be significantly related with reports of perceived stress (**Table 2**), the contribution did not reach significance in the regression analyses. In contrast, the contribution provided by trait-emotional intelligence was observed to be substantial. An alternative explanation is that social orientation is more important and has greater effect for decision making in regular work-life settings (i.e., police investigators), compared to academic education (students).

Our specific expectation that higher reports of self-monitoring (SMS) would relate to less perceived stress was not supported. These results stand in contrast to findings that relate SMS to constructive performance and work-life success (e.g., Day et al., 2002). Future research should try to better understand *how* self-monitoring tendencies affect the outcome of decision making in different domains.

Furthermore, Machiavellian tendencies were found to be positively correlated with perceived stress in study 1. Previous research has reported that stress (experimentally induced acute stress and/or naturally occurring stress in everyday life) can make people more inclined to make egoistic decisions (Dahling et al., 2009; Starcke et al., 2011). Speculatively, persons who are distrustful of others might be more inclined to experience social feedback concerning decision making as negative and threatening and negative social feedback has been found to evoke stress and impair decision making (Kassam et al., 2009). However, in the present study, Machiavellian tendencies did not provide a significant contribution in the regression analyses. Conversely, higher reports of TEI were found to be strongly associated with lower levels of perceived stress in study 2. Given that our results found support for a relation between some aspects of social orientation and perceived stress, and that social orientation can have different effects depending on the sample and the specific demands in the context targeted, further research should explore the relation between other aspects of social orientation and perceived stress.

### Time Approach

Both studies clearly supported *Hypothesis 3* stating that time approach is associated with perceived stress. Time approach was the feature of decision-making skill most prominently associated with perceived stress. In study 1, all three facets of time approach provided significant contribution to the explanation of perceived stress, in both step 3 and step 4 of the model (i.e., controlling for the contribution provided by social orientation). In study 2, the amount of variance explained by time approach in step 4 was comparable to that observed in study 1. However, when time approach was inserted in step 5 the contribution of time approach was not significant but the non-engaged time style was still a significant predictor. The negative relation between trait-emotional intelligence and the non-engaged time-style index may explain why the contribution of time approach was not significant (in step 5) when social orientation was controlled for (in step 4).

The specific expectation that reports of a non-engaged time approach would be associated with more perceived stress was supported. But the expectation that reports of an engaged time approach would be associated with less perceived stress was not. The results showed that reports of an engaged time approach (i.e., a preference for structuring one’s time, focus on the future, succumb to time restrictions, and be persistent) were related to more perceived stress. This positive relation could be explained by a possible relation between an engaged time approach and tendencies to contemplate on possible future consequences and outcomes (Ursin and Eriksen, 2010). Thus, an engaged time approach may indicate a risk for over-commitment that may lead to perceived stress.

As expected, reports of procrastination were related to perceived levels of stress in both samples. This result confirms previous research in that, in terms of self-reported stress, procrastinators may experience short-term benefits but long-term costs (Tice and Baumeister, 1997). In sum, the results clearly demonstrate that individual differences in time approach are important to consider for understanding perceived stress in work-life settings.

### Levels of Perceived Stress as an Effect of Decision-Making Skill

In the present research, levels of perceived stress were seen as a decision-making outcome. The rationale for this is that it is reasonable to see a high level of perceived stress as negative for people’s well-being and physiological health and therefore an outcome of decision making that successful decision makers should be more likely to avoid. Moreover, negative stress is per definition a response that occurs when (perceived) demands exceed (perceived) regulatory resources (e.g., Koolhaas et al., 2011). Hence, when faced with high demands to make decisions, successful decision makers should possess resources necessary in order to meet the decision-related requirements. Given that successful decision-makers are likely to be more efficient in their work (Ceschi et al., 2017), it is noteworthy that previous research has paid so little attention to how decision-making relates to stress (Santos-Ruiz et al., 2012; Starcke and Brand, 2012).

### Limitations

The present study has various limitations. For instance, we measured three individual difference variables relating to decision making taken in a broad sense and investigated their association with perceived stress. Thus, our study is correlational and the approach is limited by the fact that it does not have any process measures. However, this approach is the same as taken in previous research on decision-making competence (e.g., Bruine de Bruin et al., 2007). It would be beneficial for future research to include process measures in order to follow events between the participants’ decisions and outcomes (see e.g., Ceschi et al., 2017).

In study 1, most participants were women. This is a limitation, as it is possible that sex can have an interaction effect. Furthermore, a potential limitation of the present study is the low response rate in study 2. The response rate might indicate that the present sample could be unduly influenced by participants with an overall low level of perceived stress. Yet, rigorous information preceded the implementation of the study and explicitly highlighted the focus on perceived stress in the police–investigator profession. Therefore, it is possible that the sample consists of employees with stress-related concerns. Consequently, although the response rate is a limitation, it is not clear if, or how, this may have affected the results.

Moreover, the data collection of study 2 was performed by the use of both web-based and paper-and-pen questionnaires which could be considered a limitation. However, previous research has found that the format of web-based questionnaires does not affect the content of people’s responses and that its effects are consistent with those from studies using traditional methods (see e.g., Gosling et al., 2004; Gosling and Mason, 2015).

A further limitation of the present study is its cross-sectional design. Additionally, the measure of decision-making competence was performance/ability based whereas measures of social orientation and time approach – as well as outcome measures – were self-reports. Hence, the risk for common method bias should be acknowledged. An improvement in future research would be to use a longitudinal design. For example, initial measures of perceived stress, e.g., at the beginning of a semester (students) or after summer vacations/before reorganization (professionals) could be collected. These measures could then function as base-rates to which subsequent measures can be compared. Finally, future research should target different samples and include other types of measures than self-reports.

## Conclusion

In this study we have used a broad definition of decision making by including features that often influence the decision-making process. In work-life settings we argue that successful decision-makers need to attend to social and temporal aspects in order to meet decision-making requirements. In sum, our results suggest that the general benefits associated with decision-making competence reported by previous research (A-DMC performance, e.g., Bruine de Bruin et al., 2007) may not hold predictive validity for perceived stress in work-related contexts. In contrast, our results showed that social orientation and time approach, proposed to contribute to decision making, are associated with levels of perceived stress in work-life settings.

## Ethics Statement

This research was approved by the Regional Ethical Review Board, Gothenburg secretariat (Sweden), 2011-02-21, dnr: 071-11. Written informed consent was obtained from all participants (studies 1 and 2).

## Author Contributions

All listed authors (MG and CMA) have made substantial, direct and intellectual contribution to the work and also approved it for publication.

## Conflict of Interest Statement

The authors declare that the research was conducted in the absence of any commercial or financial relationships that could be construed as a potential conflict of interest.
