# Supplementary material for: Decision-Making Competence, Social Orientation, Time Style, and Perceived Stress
Source: Front Psychol. 2018 Apr 9;9:440. doi: 10.3389/fpsyg.2018.00440 (PMC5900026; doi:10.3389/fpsyg.2018.00440)
Supplement: Supplementary file 1 [file Presentation_1.pdf]

Supplementary material.

List of items on the A-DMC component Recognizing Social Norms (RSN), excluded in Study 1 and Study 2.

1. Do you think it is sometimes OK…
   …to smoke cigarettes?
2. Do you think it is sometimes OK…
   …to keep things you find on the street?
3. Do you think it is sometimes OK…
   …to drink and drive?[1]
4. Do you think it is sometimes OK…
   …to yell and argue to solve a conflict?
5. Do you think it is sometimes OK…
   …not to hold the door open for people?
6. Do you think it is sometimes OK…
   …not to be in time for appointments?
7. Do you think it is sometimes OK…
   …not to spend time with friends in need?

[1]This item was excluded since it showed no variance in the pilot-study

List of items on the A-DMC component Recognizing Social Norms (RSN), excluded in Study 2.

1. Do you think it is sometimes OK…
   …to steal under certain circumstances?
2. Do you think it is sometimes OK…
   …to commit a crime which could put you in jail?
3. Do you think it is sometimes OK…
   …to experiment with marijuana?

List of items on the A-DMC component Recognizing Social Norms (RSN), added in Study 2.

1. Do you think it is sometimes OK…
   …to embellish your resume when applying for a job?
2. Do you think it is sometimes OK…
   …to talk on the phone in the quiet department?
3. Do you think it is sometimes OK…
   …to leave children at the kindergarten even though they may risk to infect others?

Supplementary material.

List of items on the A-DMC component Under/Over Confidence (UOC), excluded in Study 1.

1. The grace period on your credit card is the amount of time you do not have to pay interest on outstanding payments.
2. Procrastination is worse when you work in a cluttered environment.
3. It can be instructive for children to see their parents resolve a fight.
4. Credit card companies can offer lower payments if you can come up with a lump sum settlement.
5. Contracting a sexual transmitted disease is not an automatic sign that your partner has had an affair.
6. Self-employed people pay the same amount of taxes as people who work for an employer.
7. Creating a routine is an important step in getting unpleasant things done.
8. If you get in to an auto accident, let the other person take the lead in handling the details.
9. There is no way you can negotiate a lower rate with a credit company.
10. Talking about sex helps romantic relationships.

List of items on the A-DMC component Under/Over Confidence (UOC), added in Study 1.

1. Lead pencils contain lead.
2. Vikings had horns on their helmets.
3. Snuff contains small pieces of glass.
4. You can use ketchup to polish copper.
5. There is a Muslim organization, counterpart to the Red Cross, named the Red Crescent.
6. In order to be entitled to unemployment benefit, you need to be a member of a union.
7. Venus is the Greek goddess of beauty.
8. The hair on the human head grows approximately one centimeter per month.
9. The substance Nicotine is named after a French doctor named Nicot.
10. Arachnophobia refers to the fear of dogs.
